# A Prospective Observational Cohort of Clinical Outcomes in Medical Inpatients prescribed Pharmacological Thromboprophylaxis Using Different Clinical Risk Assessment Models(COMPT RAMs)

**DOI:** 10.1038/s41598-019-54842-3

**Published:** 2019-12-04

**Authors:** Nibal Chamoun, Stephanie Matta, Sandrine Sarine Aderian, Rami Salibi, Pascale Salameh, Gaby Tayeh, Elie Haddad, Hady Ghanem

**Affiliations:** 1 Lebanese American University School of Pharmacy, Byblos, Lebanon; 2 Pharmacy Department, Lebanese American University Medical Center – Rizk Hospital, Beirut, Lebanon; 3 Department of Pulmonary/Critical Care/Internal Medicine, Lebanese American University Medical Center – Rizk Hospital, Beirut, Lebanon; 4 Lebanese University, Faculty of Pharmacy, Hadath, Lebanon; 5 Institut National de Sante Publique, Epidemiologie Clinique et Toxicologie (INSPECT-LB), Beirut, Lebanon; 6 Department of Cardiology, Lebanese American University Medical Center – Rizk Hospital, Beirut, Lebanon; 7 Department of Hematology/Oncology, Lebanese American University Medical Center – Rizk Hospital, Beirut, Lebanon

**Keywords:** Medical research, Health care

## Abstract

The Caprini and Padua venous thromboembolism (VTE) risk assessment models (RAMs) are used to assess VTE risk in surgical and in medical patients respectively. This study aims to compare the proportion of medical inpatients eligible for VTE prophylaxis using the hospital Caprini-based RAM to using the Caprini and Padua RAMs and to assess the associated clinical outcomes. In a prospective observational study, we assessed 297 adult medical inpatients for whom VTE thromboprophylaxis was initiated according to the hospital Caprini-based RAM, referred to as the Lebanese American University Medical Center RAM (LAUMC-RAM). The Padua, Caprini and IMPROVE bleeding risk scores were also assessed for all patients. Bleeding and thromboembolism were evaluated at 14 and 30 days post VTE risk assessment. Pharmacologic thromboprophylaxis was warranted in 97.6%, 99.7%, and 52.9% of patients using the Caprini-based, Caprini, and Padua RAMs respectively. The Caprini-based and Caprini RAMs were highly correlated (r = 0.873 p < 0.001) and were significantly less correlated with the Padua RAM. Major and overall bleeding occurred in 1.4% and 9.2% respectively. VTE was reported in 0.4% with no VTE related mortality. In hospitalized medical patients, the Caprini-based RAM can accurately distinguish low and high VTE risk without resulting in increased risk of bleeding.

## Introduction

In the absence of thromboprophylaxis, the risk of deep vein thrombosis (DVT) ranges from 8% in general medical patients and can increase up to 56% in stroke patients. Death occurs in about 6% of DVT and 12% of pulmonary embolism (PE) cases within one month of diagnosis^1^. In the presence of thromboprophylaxis, the rate of venous thromboembolism (VTE) in medical patients has been reported to occur between 3% to 5.5%^2,3^. The incidence of DVT and PE was significantly reduced by 56% and 58% respectively with the use of heparins as compared to placebo, without a significant increase in major bleeding or deaths^4^.

Despite the efforts to provide thromboprophylaxis to patients at risk, there is ample room for improvement. Studies have shown that 42% of medical patients are at risk of VTE of which only 40% are receiving prophylaxis^5^. Regional data shows that up to 99% of medical patients in Lebanon are at a high risk for VTE. Despite this high rate, there were lower rates of American College of Chest Physicians (ACCP) guidelines compliance in Lebanon (36.9%) compared to the international data (50.2%)^5,6^.

All hospitalized patients should have their VTE risk status determined and prophylactic measures instituted if deemed appropriate. In 2012, the ACCP Guidelines provided separate recommendations for surgical and medical patients, recommending the Caprini score for non-orthopedic surgery patients and the Padua score for medical patients^7–9^. Although the Padua score has been validated in a cohort of medical patients, it does not include all risk factors taken into consideration when assessing a patient’s VTE risk in clinical practice^10^. The idea of having an evidence based VTE RAM that could be applied to both medical and surgical patients is appealing to increase compliance with VTE risk evaluation and treatment initiation if warranted^11^.

Some institutions have adopted tailored VTE RAMs according to regional and center specific practices, taking into consideration risk factors identified by international guidelines^12^. The current venous thromboembolism risk assessment score implemented at our hospital, the Lebanese American University Medical Center (LAUMC), is used to assess the need for DVT prophylaxis in both surgical and medical patients. In fact, several hospitals in Lebanon have adopted this score that was recommended by a VTE prevention awareness campaign. This score was based on the Caprini RAM, which was initially developed in medical and surgical patients and then extensively validated in surgical patients^13–16^. Slightly different cut offs for the Caprini risk score and corresponding treatment options are published in the literature^9,13,17^. In the hospital version of the Caprini based RAM, the LAUMC-RAM, pharmacological thromboprophylaxis is recommended in patients with a score ≥2, whereas the Chest guidelines recommend the initiation at a score of 3–4^9^. This cut off to initiate pharmacolgical prophylaxis at a Caprini score of ≥2 has been previously utilized by others^9,13,17^.

In most recent years, validation studies of the caprini score in medical patients have yielded conflicting results^11,18–21^. In the current prospective study we used our institution-specific, Caprini-based, RAM (LAUMC-RAM) on a cohort of medical patients and compared it to both the Padua and Caprini RAM in its ability to accurately risk stratify non-surgical patients into low and high VTE risk groups.

The aim of the study was to evaluate the use of a Caprini based VTE risk assessment model in medical inpatients and to report the associated clinical outcomes. The primary objective was to compare the proportion of patients considered at high risk for VTE (and thus eligible for VTE prophylaxis) according to 2 published risk assessment models (the Padua prediction and the Caprini scores) and to compare these results with the proportion identified by the LAUMC-RAM for VTE.

The secondary objectives included the characterization of patients’ bleeding risk scores, according to the IMPROVE bleeding risk score, in whom thromboprophylaxis was prescribed, and to identify the occurrence of clinical endpoints including symptomatic VTE at 30 days, bleeding at 14 days, and mortality throughout follow up. Other secondary endpoints included the concordance of the institutional Caprini-based RAM with the guideline recommended risk assessment models.

## Methods

The prospective validation study was conducted between October 2015 and October 2017 at the LAUMC a university medical center in Beirut, Lebanon. Eligible patients were identified by screening all non-surgical patients admitted to the medical floor >18 years old, for whom both pharmacological prophyalxis was initiated and a hospital thrombosis risk factor assessment form was completed by the admitting medical team within the first 24–48 hours. Patients on anticoagulant treatment or those with an indication for full dose therapeutic anticoagulation upon hospital admission were excluded.

In parallel to the data collection of the thrombosis risk factor assessment form completed by the medical team and available in the chart, a study investigator prospectively completed three other scores on admission: the Padua RAM, Caprini RAM and the Improve bleeding risk score which were not shared with the patient or the treating medical team in order not to influence the physicians’ clinical practice. The investigators prospectively followed up on the occurrence of bleeding and thrombotic events throughout the hospitalization period and at 14 and 30 days post VTE risk assessment respectively. If patients were discharged before the follow up was due, telephone calls were conducted to inquire about any signs or symptoms of bleeding or thrombotic events. Death throughout the duration of the study was also collected from the medical records and follow up telephone calls. Medical data including laboratory investigations and study outcomes were collected after obtaining patients’ signed informed consent. In the event that a participant was not in a condition to provide consent, the legal representative or next of kin was asked to consent on behalf of the patient. Patients who chose not to enroll in the study continued to receive thromboprophylaxis at the discretion of their treating physician. The study was performed in accordance with the ethical standards as depicted in the 1964 Declaration of Helsinki and its later amendments. The institutional review board (IRB) of the Lebanese American University (LAU) approved this study.

The LAUMC-RAM used a point-scoring system with 41 risk factors. Patients were classified as having a low (Caprini risk Score 0–1), moderate (Caprini risk score 2), high (Caprini risk score 3–4) or highest (Caprini risk score ≥5) risk of VTE. In comparison to the Caprini RAM, the LAUMC-RAM allocates different cutoffs and risk points for BMI, attributing higher VTE risk with higher BMI, BMI ≥30 & <40 = 1 point, BMI ≥40 & <50 = 2 points and BMI ≥50 = 3 points. In contrast, the Caprini RAM attributes 1 point to a BMI ≥25. The points allocated to surgery depending on the duration of surgery also differ between the 2 scores. The LAUMC-RAM attributes increasing points from 1, 2, 3, and 5 to surgeries of 0–5, 60–119, 120–179, and ≥180 minutes respectively. The LAUMC-RAM also differentiates between history of cancer or current cancer whereas Caprini RAM allocates 2 points regardless if the patient has a current cancer or a history of cancer. The LAUMC-RAM does not include the following risk factors: History of unexpected stillborn infant, recurrent spontaneous abortions (≥3), premature birth with toxemia or growth restricted infant. Additional risk factors considered by the LAUMC RAM are: nephrotic syndrome, tobacco use, collagen vascular disorder, and infusion of venotoxic solutions, each of which is attributed to 1 point and ICU admission attributed to a risk of 2 points (Appendix 1).

The Caprini score was calculated to be able to compare between the Caprini and the LAUMC-RAM score. The Caprini model has 40 risk factors^13^. We also prospectively collected data from the medical records to complete the Padua Prediction Score that includes 11 risk factors. Patients were classified as having a high (Padua Prediction Score ≥4) or low (Padua Prediction Score <4) risk of VTE^8^. Bleeding risk score was also calculated based on the IMPROVE bleeding risk score that assesses 11 factors. Bleeding risk score (BRS) ≥7 points was associated with a higher cumulative incidence of major bleeding within 14 days^22^.

The primary endpoint was the percentage of high risk VTE patients eligible for VTE prophylaxis and the concordance of the institutional Caprini-based RAM with the guideline recommended risk assessment models. The secondary endpoints were the rates of clinical endpoints including symptomatic VTE at 30 days, bleeding at 14 days post VTE assessment and mortality throughout follow up. Thrombotic events were defined as arterial or venous and further described by location: cerebral, lower extremity proximal deep venous thrombosis, pulmonary, upper extremity deep venous thrombosis, mesenteric, lower extremity distal deep venous thrombosis, coronary, line related or other. A bleeding event was defined as major if: it was overt associated with a fall in hemoglobin level of ≥2 g/dL and associated with the requirement of at least two units of blood; it was retroperitoneal, spinal or intracranial; or it occurred in a critical organ or contributed to death^8,23^. Non-major bleedings (NMB) were considered as episodes that did not qualify as major.

### Statistical methods

Data was entered and analyzed using SPSS version 22.0. For bivariate analysis, we used the Students T test for comparing the means between two groups, the Chi-square test for comparing 2 or more percentages, and the Fisher exact test whenever it was not possible to use the Chi-square test (cells containing expected values lower than 5). To estimate the association between two quantitative variables, we used the Pearson correlation coefficient; the Goodman-Kruskall gamma was calculated to assess associations between ordinal variables and the intraclass correlation coefficient (ICC) measure to assess the concordance between two quantitative variables. For the bleeding outcome, we conducted a logistic regression using a forward method. In all cases, a p-value lower than 0.05 was considered significant.

## Results

This study included 297 patients admitted to medical wards who received pharmacological thromboprophylaxis. Table 1 lists patient baseline characteristics on admission. The mean length of stay was 8.62 days, SD 23.62 and the average number of pharmacological thromboprophylaxis doses received were 7.15, SD 7.89. The majority of patients 268/297 (90.5%) were evaluated for VTE prophylaxis within 24 hours. Telephone follow up was complete for 282 (94.4%) and 267 (89.9%) patients at day 14 and 30 respectively.

**Table 1:** Baseline Characteristics.

	(N = 297)
Age (Years), mean ± SD	72.5 ± 13.4
Weight (kg), mean ± SD	70.3 ± 16.5
BMI (kg/m^2^), mean ± SD	26.0 ± 5.6
Platelets, mean ± SD	269.3 ± 123.3
**Gender**	
Males, **N** (**%**)	139 (46.8)
Females, **N** (**%**)	158 (53.2)
**Admission Diagnosis, N (%)**	
Cardiovascular	32 (10.77)
Endocrine	5 (1.68)
Gastrointestinal	28 (9.43)
Hematologic	5 (1.68)
Infectious diseases (non-pulmonary)	27 (9.09)
Neurological	16 (5.39)
Oncologic	57 (19.19)
Pulmonary (non-infectious)	44 (14.8)
Pulmonary (infectious)	43 (14.5)
Renal	7 (2.36)
Others	33 (11.11)
Smoker	92 (31)
Cancer	114 (38.5)
Previous VTE	3 (1)
Hypertension	188 (63.3)
Dyslipidemia	101 (34)
CHF	38 (12.8)
Asthma	13 (4.4)
COPD	36 (12.1)
GERD	11 (3.7)
DVT	3 (1)
CKD	38 (12.8)
DM	100 (33.8)

BMI = body mass index, VTE = venous thromboembolism, CHF = congestive heart failure, COPD = chronic obstructive pulmonary disease, GERD = gastro esophageal reflux disease, DVT = deep vein thrombosis, CKD = chronic kidney disease, DM = diabetes mellitus.

### Characterization of VTE and Bleeding Risk using different clinical risk assessment scores

Among all 297 patients, only 21/297 (7.1%) were found to have an elevated bleeding risk with an improve bleeding risk Score ≥7. Using the LAUMC-RAM score resulted in 290/297 (97.6%) of the patients being classified as moderate to high risk whereas using the Padua, only 157/297 (52.9%) fell into the high-risk category warranting pharmacological thromboprophylaxis. Both the LAUMC-RAM and the Caprini RAMs resulted in similar categorization classifying patients into the moderate to high risk category at 290 (97.6%) and 296 (99.7%) respectively.

### Comparison of the clinical RAMs

In fact, the hospital Caprini and the Caprini RAMs were highly correlated r = 0.873 p < 0.001. The Padua RAM was significantly less correlated with the other RAMs, r = 0.504 with the Caprini and r = 0.550 with the LAUMC-RAM, p > 0.001. The Intra Class Correlation Coefficient (ICC) also confirmed the reliability of the correlation between the hospital and Caprini RAMs, ICC 0.932 [0.915;0.946], p < 0.001. The LAUMC-RAM demonstrated a very good agreement with the Caprini RAM, Gamma = 0.952 (p = 0.016), with a slight under classification of the VTE risk in comparison to Caprini (Table 2). No agreement was demonstrated between the Padua and each of the Caprini and the LAUMC-RAM, Gamma = 0.573 (p = 0.081) and Gamma = 0.677 (p = 0.065), respectively. (Tables 3 and 4).

**Table 2:** Comparison between LAUMC-RAM and Caprini categories.

Caprini Categories		LAUMC-RAM Categories				Total
		Low risk	Moderate risk	High risk	Very high risk	
Low risk	N (% of total)	1 (0.3)	0 (0)	0 (0)	0 (0)	1 (0.3)
Moderate risk	N (% of total)	6 (2.0)	10 (3.4)	1 (0.3)	0 (0)	17 (5.7)
High risk	N (% of total)	0 (0)	11 (3.7)	67 (22.6)	15 (5.1)	93 (31.3)
Very high risk	N (% of total)	0 (0)	0 (0)	28 (9.4)	158 (53.2)	186 (62.6)
Total	N (% of total)	7 (2.4)	21 (7.1)	96 (32.3)	173 (58.2)	297 (100)

**Table 3:** Comparison between Padua and Caprini categories.

		Padua Categories		Total
		Low risk	High risk	
**Caprini Categories**				
Low risk	N (% of total)	1 (0.3)	0 (0)	1 (0.3)
Moderate risk	N (% of total)	17 (5.7)	0 (0)	17 (5.7)
High risk	N (% of total)	55 (18.5)	38 (12.8)	93 (31.3)
Very high risk	N (% of total)	67 (22.6)	119 (40.1)	186 (62.6)
Total	N (% of total)	140 (47.1)	157 (52.9)	297 (100)

**Table 4:** Comparison between Padua and LAUMC-RAM categories.

		Padua Categories		Total
		Low risk	High risk	
**LAUMC- RAM Categories**				
Low risk	N (% of total)	7 (2.4)	0 (0)	7 (2.4)
Moderate risk	N (% of total)	21 (7.1)	0 (0)	21 (7.1)
High risk	N (% of total)	57 (19.2)	39 (13.1)	96 (32.3)
Very high risk	N (% of total)	55 (18.5)	118 (39.7)	173 (58.2)
Total	N (% of total)	140 (47.1)	157 (52.9)	297 (100)

### Clinical outcomes

Only one patient experienced a VTE 1/267 (0.4%). The DVT occurred on day 11 of hospitalization in a patient who was classified as high risk according to Padua, and very high risk according to both the LAUMC-RAM and Caprini scores.

Bleeding occurred in 26/282 (9.2%) of patients which included 4 major bleeds and 24 non-major bleeds. Some patients experienced both a major and non-major bleed. The 4 major bleeds included 1 retroperitoneal, 2 leading to a blood transfusion and 1 hemoglobin drop of more than 2. Non-major bleeds included 1 hematuria, 10 bruises, 12 cases of epistaxis, 4 rectal bleeds and 1 bleeding from the gum. Some patients experienced more than 1 non-major bleed. In the bivariate analysis, being on antiplatelets or anticoagulants was significantly associated with higher rates of overall bleeding which occurred in 15 (5.5%) vs 11 (100%) p < 0.001, major bleeding 2 (0.7%) vs 2 (18.2%), p = 0.008 and NMB 14 (5.2%)vs 10 (90.9%), p < 0.001, as compared to patients not receiving antiplatelets or anticoagulants during the bleeding events. The use of antiplatelets or anticoagulants during the bleeding events, diagnosed during follow up, were significantly associated with major or non-major bleeding, OR (29.9); CI (3.8–236.7) and OR (183.6); CI (21.9–1536.8) respectively. In the bivariate analysis, cancer as a comorbidity was not associated with the rates of bleeding.

Upon analysis of all patients who were classified as low risk according to the Padua scores, there was no difference in the incidence of overall bleeding regardless of the Caprini risk classification. When analyzed according to Caprini scores the rates were 0 (0%) in low risk Caprini, in moderate risk 4 (23.5%), high risk 4 (7.5%) and very high risk 5 (7.8%), p = 0.223. Only one patient who would have been otherwise classified as low risk according to Padua and moderate risk according to Caprini, experienced a major bleed.

Among the NMB, all patients who were classified as low risk Padua were classified as moderate to high risk Caprini; however, the Caprini classification was not associated with an increase in NMB, p = 0.590. According to the Caprini classification, NMB occurred in 0 (0%) low risk score, 3 (17.6%) moderate risk score, 4 (7.5%) high risk score and 5 (7.8%) very high risk score, p = 0.590. Consistent results were shown when the Padua low risk patients were analyzed according to the LAUMC-RAM. Regardless of the Caprini classification, there was no difference in overall bleeding, p = 0.816 (Table 5). According to the LAUMC-RAM classification, NMB occurred in 1 (14.3%) low risk score, 2 (9.5%) moderate risk score, 5 (9.1%) high risk score and 4 (7.7%) very high risk score, p = 0.949. Only one patient who would have been otherwise classified as low risk according to Padua fell into the moderate risk category according to LAUMC-RAM and experienced a major bleed. The higher improve bleeding score was not associated with a statistically significant difference in the rate of bleeding. Among patients with high risk of bleeding, none had bleeding regardless of their VTE RAM classification.

**Table 5:** Bleeding amongst Padua RAM reclassified according to Caprini based RAMs.

PADUA	Low				High		Total				Total
	N (%) = 135				N (%) = 147		N = 282*				N = 282*
LAUMC-RAM	Low N (%)	Moderate N (%)	High N (%)	Very high N (%)	High N (%)	Very high N (%)	Low N (%)	Moderate N (%)	High N (%)	Very high N (%)	N (%)
Overall bleeding	1 (14.3	3 (14.3)	5 (9.1)	4 (7.7)	3 (8.3)	10 (9)	1 (14.3)	3 (14.3)	8 (8.8)	14 (8.6)	26 (9.2)
	p = 0.816				p = 1.00		P = 0.812				
NMB	1 (14.3)	2 (9.5)	5 (9.1)	4 (7.7)	3 (8.3)	9 (8.1)	1 (14.3)	2 (9.5)	8 (8.8)	13 (8.0)	24 (8.5)
	p = 0.949				P = 1.00		P = 0.941				
Major bleeding	0	1 (4.8)	0	0	0	3 (2.7)	0	1 (4.8)	0	3 (1.8)	4 (1.4)
	p = 0.140				P = 1.00		P = 0.348				
**CAPRINI**	**Low**	**Moderate**	**High**	**Very high**	**High**	**Very high**	**Low**	**Moderate**	**High**	**Very high**	**Total**
Overall bleeding	0 (0)	4 (23.5)	4 (7.5)	5 (7.8)	2 (6.1)	11 (9.6)	0 (0)	4 (23.5)	6 (7)	16 (9)	26 (9.2)
	p = 0.223				P = 0.733		P = 0.188				
NMB	0 (0)	3 (17.6)	4 (7.5)	5 (7.8)	2 (6.1)	10 (8.8)	0	3 (17.6)	6 (7)	15 (8.4)	24 (8.5)
	p = 0.590				P = 1.00		P = 0.536				
Major bleeding	0	1 (5.9)	0	0	0	3 (2.6)	0	1 (5.9)	0	3 (1.7)	4 (1.4)
	p = 0.072				P = 1.00		P = 0.288				

*The total number of patients doesn’t equal 297 due to missing follow up.

Mortality occurred in 20 (6.97%) of the patients. None of the mortality events were reported to be VTE related mortality. Amongst the VTE RAMs, the LAUMC-RAM was not associated with mortality whereas only the Padua RAM was. A high Padua score >4 was associated with mortality, (3) 2.1% vs (17) 10.8%, p = 0.003. Among patients who were classified as low risk using Padua, there was no increase in mortality when analyzed according to the Caprini risk score, with rates of mortality of 1 (1.8%) in high risk Caprini and 2 (3%) in very high risk Caprini, and non in the moderate risk Caprini, p = 0.885. Similar findings were observed when analyzed according to the LAUMC-RAM, none in the moderate risk Caprini, 1 (1.8%) in high risk Caprini and 2 (3.6%) in very high risk Caprini, p = 0.744. Although no multivariate analysis was conducted to assess the factors associated with mortality, it is important to note that 13 (65%) of the patients who died had a comorbidity of cancer.

We analyzed our patients in accordance with the risk levels and corresponding treatment in order to assess compliance with ACCP recommendations. 93 (31.3%) had a Caprini score ≥3 warranting optional pharmacological prophylaxis and 186 (62.6%) ≥5 warranting pharmacological prophylaxis. According to the ACCP Caprini stratification, a total of 279 (94%) patients, including high and very high risk Caprini, received pharmacological prophylaxis. According to the ACCP recommendations for medical inpatients, only 157 (52.9%) of patients should have received pharmacological prophylaxis.

## Discussion

With slight modification of the Caprini score, the total risk scores between the LAUMC-RAM and the Caprini were similar. Concordance between the LAUMC-RAM and Caprini score translated into similar prescription patterns of thromboprophylaxis. However, with the use of the hospital version of the Caprini prediction score almost twice as many patients in our population were labelled as being at a moderate to high risk of VTE as compared with the Padua prediction score.

The Caprini score has shown to have a higher sensitivity as compared to the Padua score mostly due to the comprehensive list of risk factors in the Caprini RAM. In contrast, the Padua score has demonstrated a higher specificity, therefore the capability of not recommending prophylaxis when prophylaxis isn’t warranted^11,20^. Using the Caprini-based score in our practice allowed us to prescribe prophylaxis to more patients who would’ve otherwise been excluded according to the Padua score. In the era of low thromboprophylaxis prescription rates, it might be reasonable to focus on sensitivity and sacrifice specificity in order to be able to provide more patients with prophylaxis^11^. A retrospective validation of the Caprini model in Chinese hospitalized patients for primarily non-surgical reasons showed that patients were less likely to be misclassified as low risk and develop a VTE if assessed using the Caprini in comparison to the Padua. Among the patients who had developed a VTE, 75 (21.6%) versus 266 (76.7%) were classified as low risk according to the Caprini and Padua scores respectively, p < 0.0001^24^. There is however limited data to suggest that the Caprini RAM is unable to identify medical patients who benefit from thromboprophylaxis despite a linear association between the Caprini score and VTE risk^18^. This data has been criticized for its observational study design and failure to report non-pharmacological prophylaxis in the control group^25^.

Although our study did not have a control arm, there was no increase in bleeding noted among patients who were classified as low risk Padua and yet received prophylaxis due to their Caprini-based score. An external validation of the Improve bleeding RAM reported that around 20 percent of medical patients are classified as high-risk of bleeding and would therefore not benefit from pharmacological prophylaxis^26^. It is important to note that only 7.1% of our study population were at a high risk of bleeding and were still prescribed pharmacological thromboprophylaxis.

The rate of major bleeding (1.4%) reported in our study was comparable to rates reported in the literature^22,23,26^. In contrast, the overall bleeding rate (9.2%) was slightly higher than the rates reported in the IMPROVE bleeding risk trials^22,27^. In the original IMPROVE trials, the cumulative incidence of bleeding occurred in 3.2% of patients with 1.2% classified as major and 2.1% classified as clinically relevant non-major^22^. In the 2016 validation trial, any bleeding defined as major bleeding or non-major bleeding was reported in 2.7% of the patients with 1.9% being major and 0.8% minor^23^. In the largest validation cohort of the IMPROVE investigators, any bleeding defined as major bleeding or non-major bleeding was reported in 2.6% of the patients with 1.8% being major and 1.6% NMB^25^. Of note, the definition of major bleeding used in our study was consistent with the definition used across the IMPROVE validation studies, whereas the definition of non-major bleeds in our study was more inclusive as compared to the Improve trials^22,23,25^. In the LIFENOX study, that used similar bleeding definitions as our study, the combined rates of all bleeding events were higher in the enoxaparin group occurring in 91 (2.2%) in the enoxaparin group as compared to 60 (1.5%) in the placebo group (risk ratio, 1.5; 95% CI, 1.1 to 2.1), p = 0.01. There was no difference in the rate of major bleeding and there was also no difference in the rate of clinically relevant non major bleeding between enoxaparin and placebo. The difference in overall bleeding seemed to be driven by a difference in minor bleeding events^27^. In this study, any bleeding noted in the chart or reported by the patient, which did not qualify as a major bleed, was considered as a non-major bleed. The more inclusive definition as well as the prospective follow up telephone calls along with the chart review may have increased the reporting of minor bleeds and could have potentially contributed to the observed higher rate of overall bleeding. Similar overall rates of bleeding of 8.6% in patients prescribed thromboprophylaxis have been reported in a study that looked at total bleeding events without having a stringent definition for bleeding^28^. Although cancer did not affect the rate of bleeding in this study, previous literature has showed higher rates of bleeding in cancer patients as compared to non-cancer patients receiving thromboprophylaxis^29,30^.

The use of concomitant antiplatelets or anticoagulants during the 14 day follow up were highly associated with bleeding events. Bleeding in the presence of antiplatelets while on thromboprophylaxis has been observed in previous literature^31^. This study confirmed concomitant use of antiplatelets as an independent risk factor associated with bleeding in thromboprophylaxis.

Pharmacological VTE prophylaxis has not shown to significantly affect bleeding rates^31–34^. Data from prospective randomized clinical trials, meta-analysis and guidelines report no significant differences in the rates of major bleeding between pharmacological thromboprophylaxis as compared to placebo^32,35^. However, a recent systematic review of heparin, including unfractionated heparin and low molecular weight heparin (LMWH), for the prevention of VTE in acutely ill medical patients showed an increase in bleeding associated with heparin in comparison to placebo in terms of major and minor bleeding, OR 1.83; 95% CI 1.09–3.07; P = 0.02 and OR 1.61; 95% CI 1.26–2.08; P = 0.0002 respectively^36^. The IMPROVE trials showed that pharmacologic and mechanical VTE prophylaxis were associated with a statistically significant increase in bleeding, however this significance was not seen with LMWH or aspirin^22^. The rates of major bleeding reported in this study were comparable to other studies. This could be partly due to the fact that the majority of patients 83.3% were prescribed enoxaparin for thromboprophylaxis as compared to 11.7% who received unfractionated heparin.

It would be convenient if existing VTE RAMs could also be used as an index for comorbidities, disease severity and prognosis^11,37^. Inconsistent findings have been reported in the literature with regards to the Padua and Caprini serving as a mortality prediction scores^11,37^. Our results were consistent with prior studies that linked the Padua score to mortality^37^.

Almost forty percent of our study population had cancer. Based on the 2014 American Society of Clinical Oncology (ASCO) guidelines for VTE prophylaxis in oncology patients, most medical inpatients with active malignancy and no contraindications should receive thromboprophylaxis. The thromboprophylaxis recommendations for patients admitted for treatment with chemotherapy or minor procedures remains inconclusive^38^. This is consistent with the risk assessment scores of both Padua and Caprini whereby a patient with active cancer and one additional risk factor such as reduced mobility is classified as high risk for VTE and should be administered pharmacologic thromboprophylaxis. In contrast, the LAUMC-RAM automatically placed active cancer patients in the high risk category requiring thromboprophylaxis regardless of the presence of additional VTE risk factors. A study comparing Caprini and Padua RAMs was conducted in a tertiary oncology department in Poland and revealed that both RAMs were inconsistent in the VTE risk assessment of cancer inpatients, with the Caprini RAM being preferred over Padua in this setting. This study also reported an overuse of pharmacologic thromboprophylaxis in this patient population without a significant increase in bleeding risk^39^. Another comparative study in a Chinese inpatient population showed that Padua and Caprini RAMs had a similar predictive value in cancer patients with Caprini having a higher predictive value in non-cancer patients^40^. Therefore, Caprini RAM can be appropriately utilized in medical oncology inpatients in the absence of better validated tools in this setting.

A notable strength in this study was that we used the IMPROVE BRS to characterize the population’s bleeding risk in whom thromboprophylaxis was prescribed according to a Caprini-based RAM. The high rate of prospective follow up on patient reported clinical outcomes also adds to the strength of the study since patients are less likely to forget if they experienced any undesirable outcome.

Our study has several limitations to note. Our study did not evaluate how many eligible hospitalized medical patients received prophylaxis since we only included patients who were prescribed thromboprophylaxis. We also did not report on the number of patients who received mechanical thromboprophylaxis although we know this population is limited since there are a limited number of intermittent pneumatic compression devices available at the institution. In addition, one of the main weaknesses was that the VTE diagnosis was clinical and the study sample size was relatively small, which makes it difficult to comment on the rate of VTE. Moreover, we did not perform a head-to-head comparison of VTE and bleeding rates for patients treated using the Padua RAM vs patients treated using the LAUMC-RAM score.

## Conclusion

In conclusion, the study results show that in hospitalized medical patients, the LAUMC-RAM identified more high VTE risk patients without resulting in an increased risk of bleeding as compared to the Padua RAM. These findings suggest the use of the Caprini RAM in medical patients, awaiting further prospective clinical outcome studies to validate our results.

## Supplementary information

Supplementary Information

## Data Availability

The datasets generated during and/or analyzed during the current study are available from the corresponding author on reasonable request.
