# Supplementary material for: A Prospective Observational Cohort of Clinical Outcomes in Medical Inpatients prescribed Pharmacological Thromboprophylaxis Using Different Clinical Risk Assessment Models(COMPT RAMs)
Source: Sci Rep. 2019 Dec 4;9:18366. doi: 10.1038/s41598-019-54842-3 (PMC6892868; doi:10.1038/s41598-019-54842-3)
Supplement: Supplementary file 1 — Supplementary Information [file 41598_2019_54842_MOESM1_ESM.pdf]

Appendix 1. LAUMC- RAM

University Medical Center

Medical Direction

# Thrombosis Risk Factor Assessment

Patient's name: ..................................................
File # ...................................... NOG: ..................
DOB: ...................................... Bed: ..................
Dr.: ...................................... Status: ..................
Admission Date: ..................................................
Coverage: ..................................................

Diagnosis 1: ______________________ Height (*cm*) _______ Weight (*kg*) _________ Discharge Date ________________

Diagnosis 2: ______________________

## Each Risk Factor Represents ① Point

- ☐ Age 41-60 years
- ☐ Minor surgery planned
- ☐ History of prior major surgery (<1 month)
- ☐ Medical patient currently at bed rest
- ☐ Varicose veins
- ☐ Acute myocardial infarction (<1 month)
- ☐ Congestive Heart Failure (<1 month)
- ☐ Sepsis (<1 month)
- ☐ Serious lung disease (such as COPD)
- ☐ History of Inflammatory Bowel Disease
- ☐ Swollen legs (current)
- ☐ Obesity (BMI > 30$Kg/m^2$)
- ☐ Leg Plaster Cast or Brace (<1 month)
- ☐ Pregnancy and postpartum (<1 month)
- ☐ Oral contraceptives or HRT
- ☐ Nephrotic Syndrome
- ☐ Tobacco use
- ☐ Collagen vascular disorder
- ☐ Infusion of Venotoxic solutions via central access
- ☐ Other risk factors: ____________________________

## Each Risk Factor Represents ② Points

- ☐ Age 60-74 years
- ☐ Major surgery (>60 minutes)
- ☐ Arthroscopic surgery (>60 minutes)
- ☐ Laparoscopic surgery (>60 minutes)
- ☐ Previous Malignancy
- ☐ Central venous access
- ☐ Morbid Obesity (BMI > 40 $Kg/m^2$)
- ☐ ICU admission (>24 hurs)

## Each Risk Factor Represents ③ Points

- ☐ Age > 75 years
- ☐ Major surgery lasting 2-3 hours
- ☐ Present cancer or chemotherapy
- ☐ Obesity (BMI > 50 $Kg/m^2$)
- ☐ History of DVT/PE
- ☐ Family History of DVT/PE
- ☐ Positive Factor V Leiden
- ☐ Congenital or acquired thrombophilia
- ☐ HIT

## Each Risk Factor Represents ⑤ Points

- ☐ Elective major lower extremity arthroplasty
- ☐ Hip, pelvis or leg fracture (<1 month)
- ☐ Stroke (<1 month)
- ☐ Multiple trauma (<1 month)
- ☐ Acute spinal cord injury (Paralysis) (<1 month)
- ☐ Major surgery lasting over 3 hours

### Exclusion Criteria for Pharmacologic Thromboprophylaxis

- ☐ Bleeding
- ☐ Hypersensitivity to UFH or LMWH
- ☐ Uncontrolled Hypertension
- ☐ Significant Renal insufficiency (CrCl < 30ml/min)
- ☐ Coagulopathy (INR > 2.5; Platelets < 60,000)
- ☐ Heparin induced Thrombocytopenia
- ☐ Recent intraocular or intracranial surgery
- ☐ Spinal tap or epidural anesthesia within 24 hours

### Exclusion Criteria for IPC

- ☐ Severe peripheral arterial disease
- ☐ Congestive Heart failure
- ☐ Acute superficial / deep vein thrombosis

Total Risk Factor Score ☐

*VTE risk and suggested prophylaxis for at-risk patients*

| Total Risk Factor Score | Incidence of DVT | Risk Level | Prophylaxis Regimen | Legend |
|---|---|---|---|---|
| 0-1 | 2% | Low Risk | No Specific measures; early ambulation | ES – Elastic Stockings |
| 2 | 10-20% | Moderate Risk | LMWH or LDUH or ES | LDUH – Low Dose |
| 3-4 | 20-40% | High Risk | LMWH or LDUH | Unfractionated Heparin |
| 5 or more | 40-80% | Highest Risk | LMWH, Fondaparinux or LDUH plus ES | LMWH – Low Molecular Weight Heparin |

►► Thromboprophylaxis Order Selected: ____________________________________________________

*Examining Physician's Signature:* ________________________________ *Date:* __________________
